# Asymmetric impact of climatic parameters on hemorrhagic fever with renal syndrome in Shandong using a nonlinear autoregressive distributed lag model

**DOI:** 10.1038/s41598-024-58023-9

**Published:** 2024-04-28

**Authors:** Yongbin Wang, Ziyue Liang, Siyu Qing, Yue Xi, Chunjie Xu, Fei Lin

**Affiliations:** 1 Department of Epidemiology and Health Statistics, School of Public Health, The First Affiliated Hospital of Xinxiang Medical University, No. 601 Jinsui Road, Hongqi District, Xinxiang, Henan Province 453003 People’s Republic of China; 2 Beijing Key Laboratory of Antimicrobial Agents/Laboratory of Pharmacology, Institute of Medicinal Biotechnology, Chinese Academy of Medical Sciences & Peking Union Medical College, Beijing, 100050 China

**Keywords:** Hemorrhagic fever with renal syndrome, Nonlinear autoregressive distributed lag model, Meteorology, Asymmetric relationships, Early forecasting, Ecological study, Diseases, Infectious diseases

## Abstract

Hemorrhagic fever with renal syndrome (HFRS) poses a major threat in Shandong. This study aimed to investigate the long- and short-term asymmetric effects of meteorological factors on HFRS and establish an early forecasting system using autoregressive distributed lag (ARDL) and nonlinear ARDL (NARDL) models. Between 2004 and 2019, HFRS exhibited a declining trend (average annual percentage change = − 9.568%, 95% CI − 16.165 to − 2.451%) with a bimodal seasonality. A long-term asymmetric influence of aggregate precipitation (AP) (Wald long-run asymmetry [WLR] = − 2.697, *P* = 0.008) and aggregate sunshine hours (ASH) (WLR = 2.561, *P* = 0.011) on HFRS was observed. Additionally, a short-term asymmetric impact of AP (Wald short-run symmetry [WSR] = − 2.419, *P* = 0.017), ASH (WSR = 2.075, *P* = 0.04), mean wind velocity (MWV) (WSR = − 4.594, *P* < 0.001), and mean relative humidity (MRH) (WSR = − 2.515, *P* = 0.013) on HFRS was identified. Also, HFRS demonstrated notable variations in response to positive and negative changes in ∆MRH(−), ∆AP(+), ∆MWV(+), and ∆ASH(−) at 0–2 month delays over the short term. In terms of forecasting, the NARDL model demonstrated lower error rates compared to ARDL. Meteorological parameters have substantial long- and short-term asymmetric and/or symmetric impacts on HFRS. Merging NARDL model with meteorological factors can enhance early warning systems and support proactive measures to mitigate the disease's impact.

## Introduction

Hemorrhagic fever with renal syndrome (HFRS) is a viral disease caused by hantaviruses, which are transmitted to humans through contact with infected rodents^1^. The disease is characterized by fever, hemorrhage, and renal failure, and can be fatal in severe cases. HFRS has been recognized as a significant public health concern in many parts of the world, particularly in Asia and Europe^1–3^, where it is endemic in certain regions^1,3,4^. While the disease is found in many countries, the prevalence of HFRS can vary widely from one region to another. This variation can be attributed to a number of factors, including differences in rodent populations, climate, and human behavior^5^. In some areas^1,3,4^, such as China, Russia, and Korea, HFRS is endemic and outbreaks occur regularly, while in other regions, such as Europe and North America, the disease is relatively rare. There is also variation in the strains of hantaviruses that are prevalent in different regions^6^. Hantaviruses are classified into different genotypes based on their genetic sequences, and each genotype is associated with specific rodent species. For example, the Hantaan virus (HTNV) is associated with the striped field mouse in Asia^1^, while the Seoul virus (SEOV) is associated with the brown rat in Europe and North America^6^. After the introduction of hantavirus into China, it adapted to various host animals, including a diverse range of rodents, shrews, and bats. Research indicates that there are over 8 suitable host species for the SEOV and 10 species of wild mouse hosts for the HTNV in China, which has currently constituted 90% of global HFRS cases^4^. Although great achievements have been made in implementing surveillance and control measures to mitigate the impact of HFRS in China^4^, most provinces, such as Shandong, Heilongjiang, Jilin, Liaoning, and Hubei, have reported endemic cases of over 20,000^4^, leading to an annualized death rate of approximately 5–10% in past years^7^, and a recent study indicated a recurring sign in HFRS morbidity owing to variants in circulating strains^8^. For this reason, recognizing the intrinsic association of potential factors with HFRS and developing an enhanced early warning system assist in comprehending the disease’s patterns and dynamics, ultimately aiding in the prevention and control of HFRS epidemic.

Climate change significantly affects the distribution and transmission dynamics of infectious diseases. The pathogens, vectors, and hosts associated with infectious diseases are highly responsive to the environments they inhabit^9–11^. Ecological barriers affect the transmission of viruses from natural or intermediate hosts to human populations, with four key factors including transmission routes, transmission probabilities, contact frequencies, and virus characteristics^12^. Climate change can weaken ecological barriers, increasing the emergence and transmission probabilities of emerging infectious diseases^12^. A recent study revealed that over 58% of infectious diseases faced by humanity worldwide have been exacerbated by climatic hazards (such as atmospheric warming, heavy precipitation, and flooding) at some point, and climatic hazards, via vector-borne transmission, contributed to increased incidence and prevalence of over 100 vector-borne diseases^13^. Under the impetus of climate change, it is estimated that by 2070, there will be at least 15,000 new instances of cross-species viral spillover. These heightened opportunities for viral sharing may increase the risk of emerging vector-borne diseases jumping from animals to humans in the next 50 years, especially in Africa and Asia^14^.

Studies have also linked climatic variables with HFRS^11,15,16^. For instance, Luo et al. indicated that a 6-month lag in mean temperature (MT) (RR = 3.05) and no lag in aggregate precipitation (AP) (RR = 2.08) had the most significant impact on HFRS in China using a generalized additive model (GAM)^17^. Chen et al.^15^ found that humidity and wind speed were correlated with the onset of HFRS, and there existed a non-linear exposure-lag-response relationship in Shenyang using a GAM. Wang et al.^16^ observed that the most influential meteorological factors for HFRS were mean temperature with a 4-month lag, mean ground temperature with a 4-month lag, and mean air pressure (MAP) with a 5-month lag in Heilongjiang using Geodetector and autoregressive integrated moving average (ARIMA) models. However, there are gaps: (1) most studies have concentrated on the effects of temperature, air pressure, rainfall, and humidity on HFRS^16,18^, with scant evidence concerning sunshine and wind’s impact. But these six meteorological factors are coexisting, and the combined exposure may have complex interactions between positive and negative changes in these factors on HFRS; (2) previous work often neglected to account for autocorrelations among dependent variables^15,18,19^, leading to potential overestimations; (3) crucially, there is an absence of research probing into the dynamic impacts of climatic changes on HFRS—understanding if increases or decreases in climatic factors lead to differing effects and how potential factors respond to changes in the short run and how these responses evolve over time are vital for comprehensive insights into HFRS transmission control. To fill these gaps, we employed the nonlinear autoregressive distributed lag (NARDL) model^20^. The choice was motivated by its advantages^20–23^: (1) it discerns both long- and short-term asymmetries between climatic variables and HFRS; (2) it offers flexibility regarding the cointegration of variables and possesses strong statistical power even with smaller sample sizes; (3) it effectively mitigates endogeneity issues among climatic variables; (4) it can automatically specify autocorrelations among variables. We hypothesize that climatic variables play a pivotal role in the transmission of HFRS both in the long and short terms, and the NARDL model by including climatic variables can improve the ability in forecasting HFRS epidemic compared to the linear autoregressive distributed lag (ARDL) model. Given that Shandong (Geographical distribution can be seen in Fig. S1) holds the distinction of being the riskiest among all HFRS-endemic provinces in China^4^, our study aims: (1) to clarify both long- and short-term asymmetric correlations between climatic variables and HFRS in Shandong using the NARDL model; (2) to ascertain if the NARDL model offers a more precise estimation of HFRS epidemic compared to the ARDL model.

## Material and methods

### HFRS data

The monthly HFRS cases from January 2004 to December 2019 in Shandong were sourced from the Data-center of China Public Health Science (DCPHS) under the Chinese CDC’s management (https://www.phsciencedata.cn/Share/en/data.jsp?id=0aeeaf46-415d-49b3-9442-df31305e669e&show=0). Concurrently, population data for the same timeframe was extracted from the Shandong Statistical Yearbook 2022 (http://tjj.shandong.gov.cn/tjnj/nj2022/zk/zk/indexch.htm). All HFRS cases were confirmed in alignment with the diagnostic criteria set by the Chinese Ministry of Health (http://www.nhc.gov.cn/wjw/s9491/wsbz.shtml). Once verified, these cases were promptly reported within 24 h by accredited institutions and professionals.

### Meteorological data

Daily climatic metrics, comprising mean temperature (MT), mean air pressure (MAP), aggregate precipitation (AP), aggregate sunshine hours (ASH), mean relative humidity (MRH), and mean wind velocity (MWV), were sourced from the National Meteorological Science Data Center (http://data.cma.cn/). Subsequently, these parameters were consolidated into a monthly format.

### Statistical analysis

The Shapiro–Wilk test informed our HFRS incidence and meteorological data presentation in summary description, expressing results as either mean ± standard deviation ($\overline{x} \pm s$) or as the median (Q_25_, Q_75_). We employed the average annual percentage change (AAPC) and seasonal relative (SR) to elucidate the trends and seasonal patterns of HFRS incidence, respectively^24,25^. Spearman’s rank (*r*_*s*_) correlation assessed the relationship between HFRS and climatic parameters. A correlation coefficient exceeding 0.9 and a variance inflation factor (VIF) surpassing 10 indicated multicollinearity among the parameters^26,27^. Such factors were thus excluded from the simultaneous NARDL and ARDL models to ensure independent effect evaluation.

The ARDL was chosen as a baseline due to its ability to navigate autocorrelations and non-stationarity while analyzing short- and long-term associations between variables^28^. Yet, given ARDL’s linear assumptions, it might not adequately capture the intricate relationship between factors when considering the asymmetric and non-linear dynamic influences of weather parameters on diseases^20,21^. Therefore, the NARDL was introduced, offering the advantage of highlighting long- and short-term asymmetric and nonlinear effects over the ARDL (a more detailed explanation of the criteria used for selecting the ARDL and NARDL models were provided in Supplementary material)^20^, which can uniquely decompose the dependent variable into its positive and negative segments of increments and decrements in independent variables. When confronted with non-linearity and asymmetry, the NARDL not only addresses autocorrelations and non-stationarity but also investigates how variables respond to changes in the short run, and how they gradually adjust and recover over the long term^20^.

The NARDL’s implementation encompasses four stages (Overall methodological flow chart is provided in Fig. S2): first, integration order testing. The NARDL can be applied irrespective of the order of integration, provided the maximum order does not exceed one^21^. Stationarity was confirmed using the augmented Dickey–Fuller (ADF) statistic^21^, and if needed, logarithmic transformations or differencing were employed to achieve this. Second, long-term asymmetric cointegration^20^. A bounds test was used to determine if there was a long-term asymmetric cointegration between variables^20,21^. If found, the Wald test explored the associated short- and long-term asymmetries. Third, effect estimation. This entails quantifying dynamic multiplier responses of the dependent variable to changes in regressors using positive and negative partial sum decompositions^20,23^. Lastly, forecasting ability assessment. The model’s predictive capability for HFRS epidemic based on weather parameters was evaluated using data from January 2004 to December 2018 as training samples and subsequent data as testing samples. Also, a sensitivity analysis was conducted, where samples from January 2004 to December 2018 were utilized for model development, and the remaining 24 samples were employed to validate the stability of the predicted outcomes. The comparison between NARDL and ARDL’s predictive capacity employed various metrics such as root mean square error (RMSE), mean absolute deviation (MAD), mean error rate (MER), and mean absolute percentage error (MAPE).

1 $${\text{MAD}} = \frac{1}{N}\sum\limits_{i = 1}^{N} {\left| {X_{i} - \hat{X}_{i} } \right|}$$

2 $${\text{RMSE}} = \sqrt {\frac{1}{N}\sum\limits_{i = 1}^{N} {\left( {X_{i} - \hat{X}_{i} } \right)^{{2}} } }$$

3 $${\text{MAPE}} = \frac{1}{N}\sum\limits_{i = 1}^{N} {\frac{{\left| {X_{i} } \right. - \left. {\hat{X}_{i} } \right|}}{{X_{i} }}}$$

4 $${\text{MER}} = \frac{{\frac{1}{N}\sum\limits_{i = 1}^{N} {\left| {X_{i} - \hat{X}_{i} } \right|} }}{{\overline{X}_{i} }}$$

The NARDL formula is represented as:

5 $$\begin{aligned} log\left( {Y_{t} } \right) & = a_{0} + \mathop \sum \limits_{i = 1}^{{p_{i} }} \varphi_{i} \log \left( {Y_{{t - p_{i} }} } \right) + \mathop \sum \limits_{i = 0}^{{q_{1} }} \delta_{1i}^{ + } x_{{t - q_{1i} }}^{ + } + \mathop \sum \limits_{i = 0}^{{q_{2} }} \delta_{1i}^{ - } x_{{t - q_{2i} }}^{ - } \\ & \;\;\; + \mathop \sum \limits_{i = 1}^{{p_{2} }} p_{2i} \Delta {\text{log}}(Y_{{t - p_{2i} }} ) + \mathop \sum \limits_{i = 0}^{{q_{3} }} \tau_{3i}^{ + } \Delta x_{{t - q_{3i} }}^{ + } \mathop \sum \limits_{i = 0}^{{q_{4} }} \tau_{4i}^{ - } \Delta x_{{t - q_{4i} }}^{ - } + a_{1} \;month + \in_{t} \\ \end{aligned}$$

where $Y_{t}$ represents HFRS cases, $x$ signifies the climatic variables, $x^{ + }$ and $x^{ - }$ are the positive and negative partial sums of increases and decreases in each climatic variable, respectively, $p$ and $q$ denote the optimal delayed orders of HFRS and weather factors, respectively, $\delta_{1i}^{ + }$ and $\delta_{1i}^{ - }$ correspond to the long-term equilibrium (this refers to the stable relationship that exists between meteorological factors and HFRS over an extended period. It captures the persistent effects of meteorological conditions on the HFRS transmission, taking into account any gradual adjustments or trends that may occur over time. By identifying this relationship, researchers can gain insights into the sustained impact of meteorological factors on HFRS, allowing for the development of targeted interventions and policies to mitigate the risk of transmission) parameters for the dependent variable, $\tau_{3i}^{ + }$ and $\tau_{3i}^{ - }$ correspond to the short-run (this refers to the immediate or temporary effects of meteorological factors on HFRS. It captures the rapid fluctuations or deviations in HFRS transmission that are attributable to sudden changes in weather conditions. Understanding this association is essential for timely response measures and forecasting, enabling public health authorities to implement proactive strategies to prevent or contain outbreaks in real-time) parameters for the dependent variable, month denotes the seasonal component, t refers to the time variable spanning from 1 to 192, and Δ refers to the first-order difference.

The maximum delay orders were set at 4 considering the 1–6-week gap from hantavirus infection to symptom onset and an additional 2-month interval from symptom onset to clinical diagnosis in China^1^. Subsequently, the determination of optimal delay orders was guided by the Akaike Information Criterion (AIC, the use of AIC in the selection is justified by its ability to: (1) balance the trade-off between model complexity and goodness of fit balance model fit and complexity. By penalizing the number of parameters in the model, AIC discourages overfitting; (2) facilitate model comparison by providing a quantitative measure of the relative quality of different models; (3) provide a solid statistical foundation for the selection process)^29^. The partial autocorrelogram (PACF) revealed correlations between current and past values within the given conditions^29^. This observation of PACF helped identify the optimal autocorrelation orders for HFRS. To account for seasonal effects, a full set of monthly dummies as fixed regressors were included in our model (which was set using the program of @expand(@month,@droplast in EViews. By doing so, an 11-month dummy parameter was produced). Furthermore, the stability of NARDL underwent verification through the use of cumulative sum (CUSUM) and CUSUM of squares (CUSUMQ) plots^20^, and the resulting errors of both models whether behaved like a white noise series (which refers to the residual series that is uncorrelated and has a constant variance) were judged by the Box-Ljung Q statistic^29^. All statistical procedures were conducted using R 4.2.0 (R Development Core Team, Vienna, Austria) and EViews 12 (IHS, Inc. USA), with a significance level at *P* ≤ 0.05 (two-sided).

### Ethics approval and consent to participate

The institutional review board of Xinxiang Medical University approved this study protocol (No: XYLL-2019072). All methods were carried out under relevant guidelines and regulations. The need for informed consent was waived by the study Ethics Committee of Xinxiang Medical University because the HFRS cases were shared anonymously and we cannot access any identifying information of the patients (available from: https://www.phsciencedata.cn/Share/).

## Results

### Statistical description

**Figure 1:**
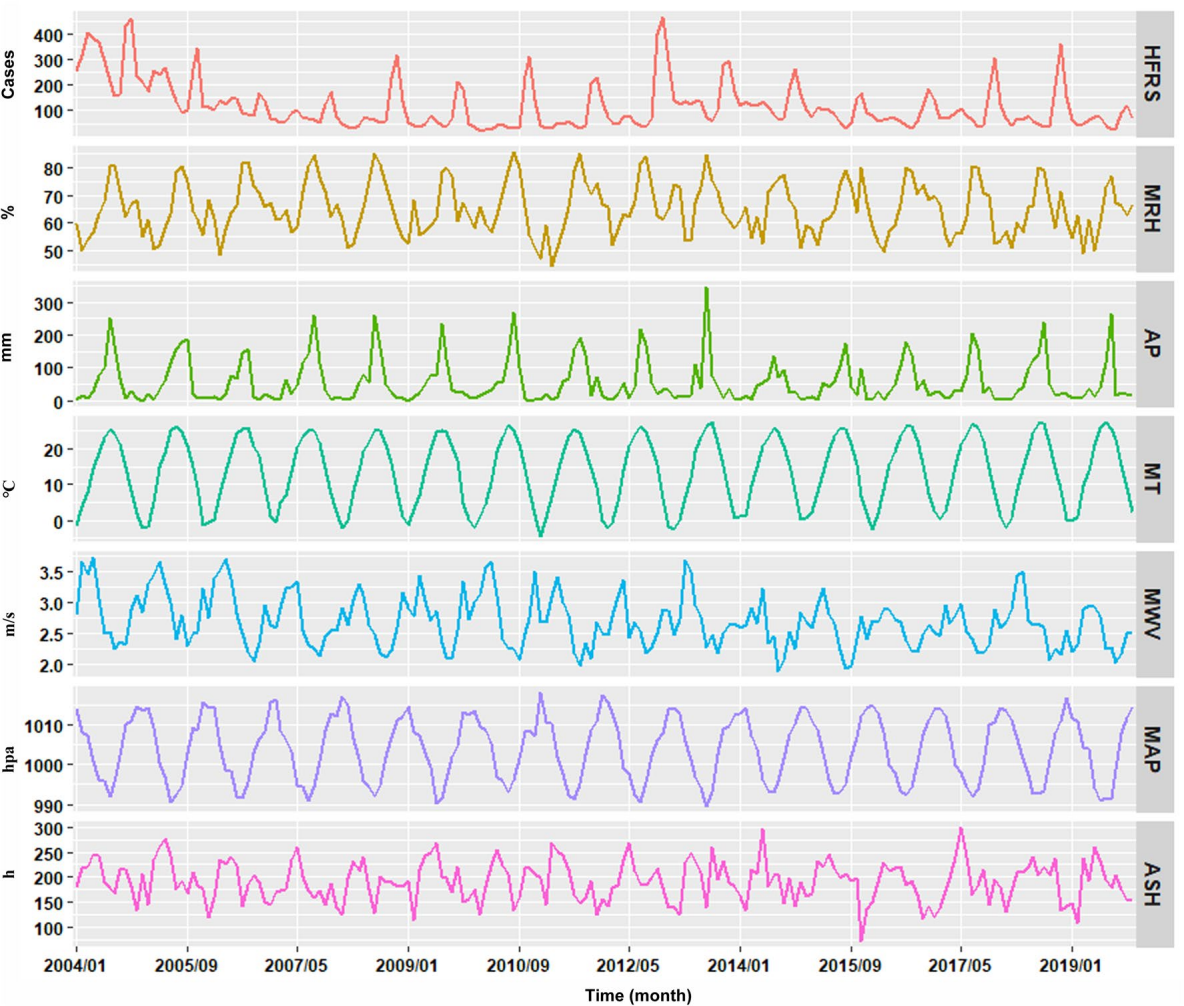
Time series plot indicating the temporal trends of the climatic factors and HFRS cases in Shandong, 2004–2019. As depicted in this figure, overall HFRS epidemic showed a downward trend. Among the meteorological factors, the MT trend was slowly increasing but the trend for others remained relatively stable during 2004–2019.

From 2004 to 2019, a total of 22,876 cases were reported, averaging 2098 annual notifications (5.708 per 100,000 individuals) and 175 monthly notifications (0.476 per 100,000 individuals). The peak year was 2004, with 4171 cases (10.931 per 100,000 population). This number was 3.727 times higher than the lowest count in 2004, which saw 1119 cases (3.261 per 100,000 population). Overall, there was a decreasing trend in HFRS incidence (AAPC = − 9.568%, 95% confidence intervals [CI] − 16.165 to − 2.451%) (Fig. 1). The decomposition SR from January to December was recorded as 0.72, 0.551, 0.643, 0.666, 0.994, 1.44, 0.779, 0.449, 0.414, 1.346, 2.629, and 1.37, suggesting a dual peak pattern, with one in June and another in October-December per year. Additionally, there seemed to be a natural cyclical pattern with a duration of about 4–7 years in HFRS incidence.

Table 1 shows that meteorological variables were also influenced by seasonality. Figure 1 indicates that HFRS trends seemed to align with those of AWV, ASH, and MAP. Conversely, the trends for HFRS and MRH, AP, along with MT appeared to be in opposition. Additionally, there was an indication of strong collinearity given *r*_*s*_ > 0.9 and VIF > 10 between MT and MAP (Table S1 and Fig. S3), meaning that these two factors should be included in different models with other variables to investigate their independent effects.

**Table 1:** Monthly mean statistics for the HFRS and climatic values in Shandong during 2004–2019.

Months	HFRS cases	MRH (%)	AP (mm)	MT (℃)	MWV (m/s)	MAP (hPa)	ASH (h)
January	97.19	60.11	5.55	− 1.36	2.66	1014.47	158.38
February	87.13	59.39	12.30	1.31	2.87	1011.63	157.58
March	103.88	53.82	14.54	7.30	3.20	1007.25	218.93
April	107.44	57.56	35.05	13.76	3.28	1001.64	229.02
May	119.25	60.23	58.84	19.75	2.98	996.86	253.18
June	100.00	66.96	75.65	23.88	2.66	993.06	218.39
July	71.31	79.15	183.00	26.17	2.38	991.81	177.97
August	160.00	80.30	175.54	25.46	2.22	994.70	188.44
September	66.63	73.23	66.61	21.28	2.14	1001.58	184.65
October	217.06	66.08	22.08	15.39	2.37	1007.77	193.76
November	269.50	64.65	26.26	7.84	2.68	1010.53	161.06
December	135.38	61.61	11.91	0.86	2.76	1017.33	157.94

### Development of the NARDL and ARDL models

**Figure 2:**
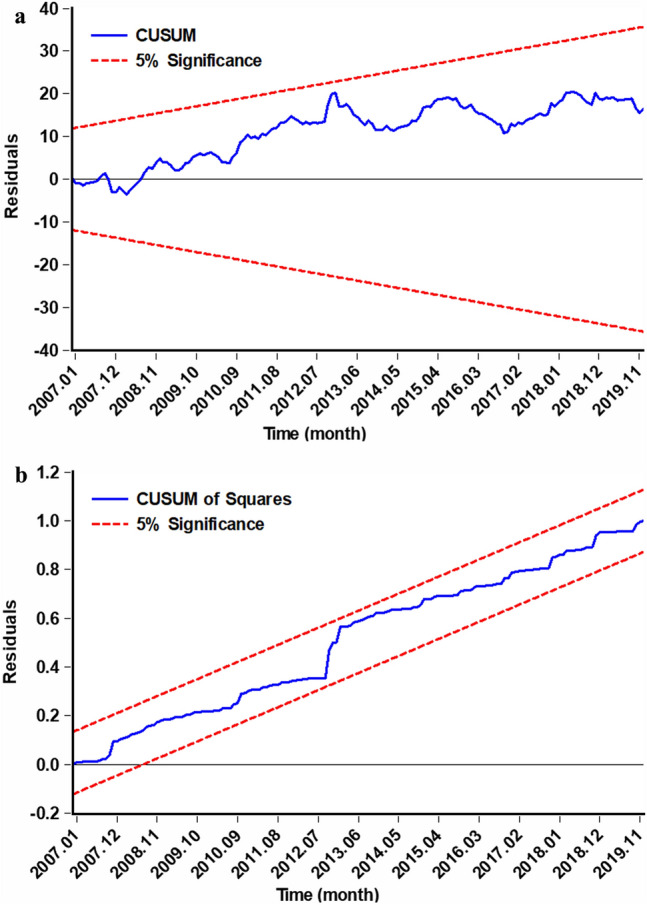
Stability test for the NARDL. (**a**) CUSUM test, (**b**) CUSUM of squares test. The CUSUM and CUSUM of squares were not beyond 95%*CI* at various time suggested the efficacy and stability of the model.

The ADF test indicated that the HFRS series (t = − 2.707, *P* = 0.007) was stationary. In contrast, the series for MT (t = 0.771, *P* = 0.879), MAP (t = − 0.857, *P* = 0.343), ASH (t = − 0.441, *P* = 0.522), MWV (t = − 1.753, *P* = 0.389), AP(t = − 0.697, *P* = 0.414), and MRH (t = − 0.224, *P* = 0.604) were non-stationary. After differencing once, all series achieved stationarity with resulting all *P* < 0.001, ensuring the requirement for modeling was met. The PACF plot highlighted the need to integrate a 1-month lag autocorrelation into the model (Fig. S4). The bounds test returned an F-value of 14.174, surpassing the critical upper bounds (I_0_ = 1.82, I_1_ = 2.99), signifying a long-term cointegration relationship between HFRS and climate variables. Subsequently, a range of NARDL models were established, Upon evaluation, the NARDL (1, 0, 2, 3, 0, 2, 0, 0, 1, 1, 0) emerged as the most suitable model, boasting the lowest AIC of 9.838 (Fig. S5). This optimal NARDL model represents parameters such as HFRS at lag 1, MRH(+) at lag 0, MRH(−) at lag 2, and so forth (Table S2). The CUSUM and CUSUMQ tests, positioned within the 5% significance levels (Fig. 2), confirmed the model’s stability. Following similar modeling procedures, the ARDL (1, 0, 0, 1, 0, 0) was identified as the best model among possible candidates (Fig. S6 and Table S3). The Box-Ljung Q statistic = 0.289 (*P* = 0.591) for the residuals from ARDL and Box-Ljung Q statistic = 2.364 (*P* = 0.124) for the residuals from NARDL indicated that both models are adequate and suitable for modeling the series.

### Long- and short-term asymmetric and symmetric impacts of weather factors on HFRS

**Figure 3:**
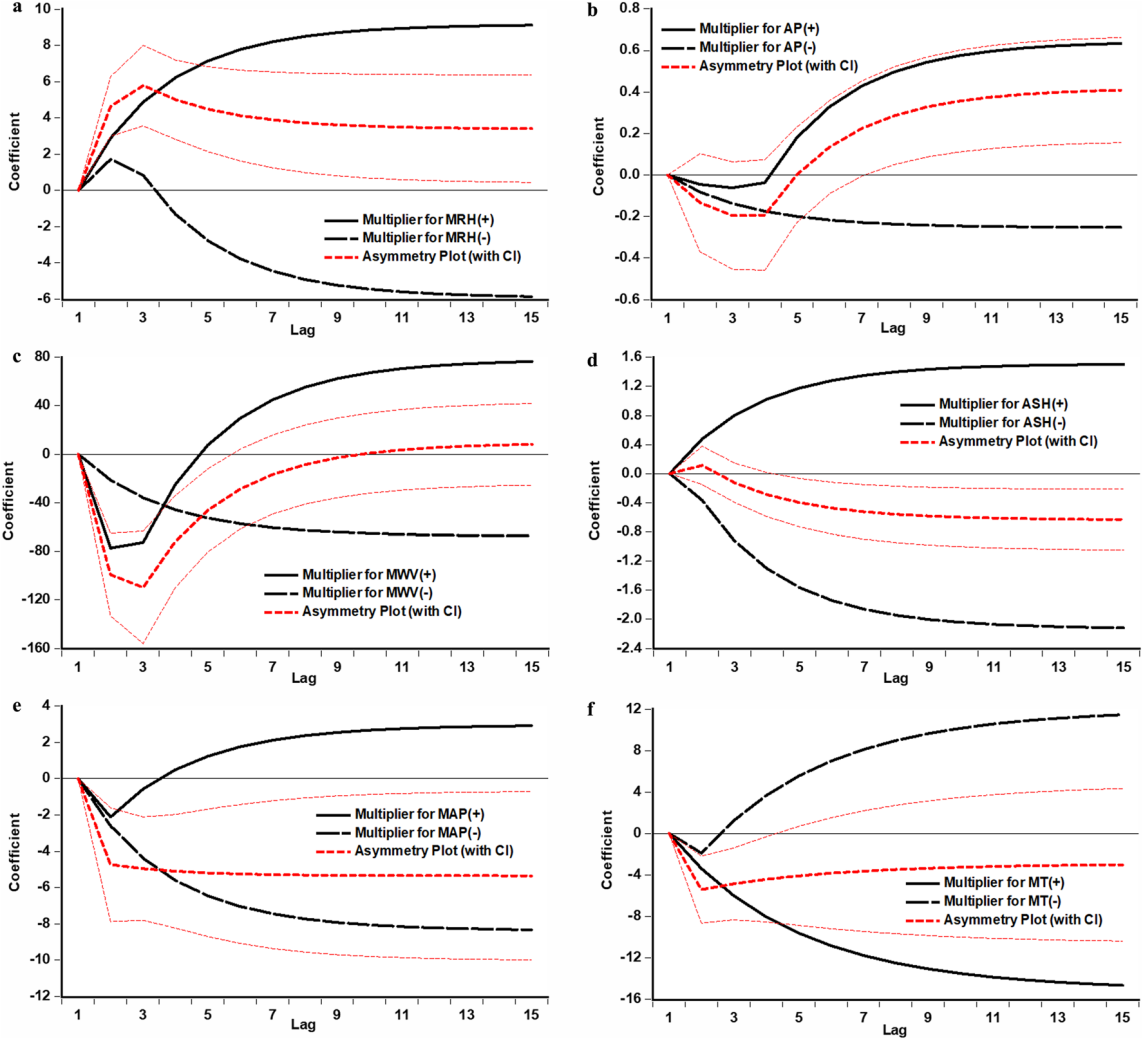
Dynamic multiplier asymmetric effects of climatic variables on HFRS. (**a**) Multiplier graph for MRH, (**b**) multiplier graph for AP, (**c**) multiplier graph for MWV, (**d**) multiplier graph for ASH, e. multiplier graph for MAP, (**f**) multiplier graph for MT. Cumulative dynamic multipliers indicated the cumulative effect of meteorological factors on the spread of HFRS over time. These multipliers help in elucidating how changes in meteorological variables influence the incidence of HFRS over a period of time. By capturing the cumulative impact of these variables, researchers can gain insights into the long-term effects of meteorology on the spread of HFRS. For instance, in (**a**), the red dashed line shows the cumulative asymmetric effect of MRH on HFRS. The coefficient is found to be positive (above 0) and statistically significant at the start of the evolution, and then the value slightly decreased (above 0) when approaching the long-run. Overall, it suggests that an increase in MRH over time is associated with a higher incidence of HFRS. In (**b**), the red dashed line changed from a negative value to a positive value, showing that a decrease in AP is first linked to a lower incidence of HFRS in the short term, but then the negative short-term asymmetric relationship would transition to a positive long-term asymmetry.

Table 2 presents both the long- and short-term asymmetry Wald tests, revealing a noticeable long-term asymmetric effect of AP and ASH on HFRS, coupled with a short-term asymmetric influence of AP, ASH, MWV, and MRH on HFRS. Table 3 shows the effect estimates, despite demonstrating no significant long-term asymmetry of MRH on HFRS, the long-run coefficients were meaningful. Notably, the coefficients of MRH and ASH were positive, while MAP has a negative coefficient with a long-run symmetric effect. Specifically, a 1% increase in MRH and a 1 h increase in ASH led to escalations in HFRS by approximately 10 (95% CI 4–16) and 2 (95% CI 1–3) cases, respectively. Conversely, a 1% decrease in MRH and a 1 h decrease in ASH resulted in increases of about 6 (95% CI  − 1–12) and 2 (95% CI 1–4) cases, respectively, in HFRS. Whereas an increase or decrease of 1 hPa in MAP declined the HFRS transmission risk by 2 (95% CI 1–3) cases. These variations in HFRS transmission risk correspond to cumulative changes in the weather parameters. Additionally, the long-run coefficients for AP and MWV were positive while MT was negative but not statistically significant. Despite this, the direction of the effects for these variables is useful. The data from Table 3 also indicates that HFRS displayed significant variations due to both positive and negative changes in weather variables, especially within short-term lags of 0–2 months. To elaborate: ∆MRH(−) and ∆AP(+) at a 1-month lag, alongside ∆MWV(+) at a 0-month lag demonstrated the most substantial negative short-term effect, with a decrement of 1% and an increment of 1 mm and 1 m/s leading to reductions in HFRS by about 2 (95% CI 1–4), 1, and 78 (95% CI 46–109) cases, respectively. While ∆ASH at a 0-month lag showed a negative short-term effect, with a decrement of 1 h contributed to a reduction in HFRS by 1 case. Likewise, there were no significant short-run coefficients in ∆MAP and ∆MT, we still captured the direction of the effects for these factors. Figure 3 visually portrays the asymmetric adjustment patterns of HFRS in adapting to the long-term equilibrium in light of positive and negative shifts in meteorological factors. This further supports the long- and short-term asymmetric and/or symmetric influences of these factors on HFRS (Fig. 3a–e). For instance, Fig. 3a shows a red dashed line that first increases and then decreases but the coefficients are always above 0, validating the negative short-term asymmetric relationship that transitions to a positive long-term asymmetry.

**Table 2:** Long- and short-term effects using the preferred NARDL and ARDL.

NARDL			ARDL		
Variable	Coefficient (95% CI)	*P*	Variable	Coefficient (95% CI)	*P*
Long-run effect			Long-run effect		
MRH(+)	9.045 (3.029, 15.061)	0.004	MRH	9.299 (− 1.935, 20.532)	0.107
MRH(−)	5.758 (− 0.342, 11.857)	0.066	AP	− 0.142 (− 1.268, 0.984)	0.805
AP(+)	0.664 (− 0.158, 1.485)	0.115	MWV	39.243 (− 129.176, 207.661)	0.649
AP(−)	0.251 (− 0.559, 1.061)	0.545	ASH	3.227 (0.795, 5.659)	0.010
MWV(+)	76.631 (− 18.92, 172.182)	0.118	MAP	− 1.727 (− 2.933, − 0.521)	0.006
MWV(−)	66.964 (− 26.386, 160.314)	0.162	MT	− 19.490 (− 62.253, 23.274)	0.373
ASH(+)	1.469 (0.262, 2.676)	0.018	Short-run effect		
ASH(−)	2.107 (0.948, 3.266)	0.001	D(MWV)	− 16.067 (− 39.285, 7.151)	0.177
MAP(+)	2.676 (− 10.066, 15.418)	0.681			
MAP(−)	8.010 (− 3.577, 19.598)	0.177			
MT(+)	− 14.661 (− 34.641, 5.32)	0.152			
MT(−)	− 11.772 (− 31.7, 8.157)	0.249			
Short-run effect					
∆MRH(−)	− 1.732 (− 3.715, 0.25)	0.089			
∆MRH(−), 1-month lag	− 1.552 (− 3.067, − 0.036)	0.047			
∆AP(+)	− 0.042 (− 0.221, 0.137)	0.644			
∆AP(+), 1-month lag	− 0.235 (− 0.428, − 0.042)	0.018			
∆AP(+), 2-month lag	− 0.198 (− 0.371, − 0.025)	0.026			
∆MWV(+)	− 77.158 (− 108.982, − 45.334)	< 0.001			
∆MWV(+), 1-month lag	− 44.755 (− 77.054, − 12.456)	0.007			
∆ASH(−)	0.357 (0.02, 0.694)	0.040			
∆MAP(+)	− 2.165 (− 5.731, 1.401)	0.236			
∆MT(−)	1.859 (− 3.526, 7.244)	0.500			

Note: MT and MAP were entered into two different models with other weather variables. Since the coefficient represents the increased or decreased HFRS cases attributable to the different meteorological factors, and thus when reporting effect sizes, it is customary to round up to the nearest whole number if there is a decimal point present in this study.

**Table 3:** Long- and short-term asymmetry results using Wald test.

Variable	Long-term asymmetry		Short-term asymmetry	
	WLR	*P*	WSR	*P*
MT	0.664	0.507	0.677	0.450
AP	− 2.697	0.008	− 2.419	0.017
ASH	2.561	0.011	2.075	0.040
MWV	0.475	0.635	− 4.594	< 0.001
MRH	− 1.810	0.072	− 2.515	0.013
MAP	1.963	0.051	− 1.190	0.236

Note: MT and MAP were entered into two different models with other weather variables.

### Forecasting ability evaluation

**Figure 4:**
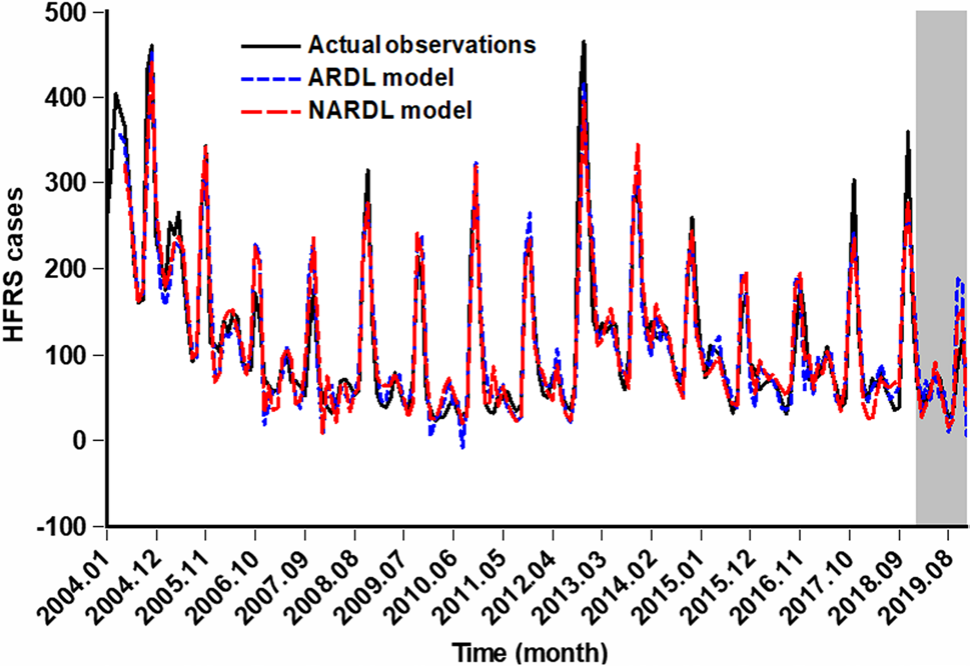
Comparison of the fitting and predictive values from the ARDL and NARDL. The results from this figure indicated that overall, the curve simulated and predicted by the NARDL model (red line) more closely matched the actual observations (black line) compared to the ARDL model, showing that NARDL model can better capture the dynamic dependence characteristics of HFRS epidemic.

Using data from January 2004 to December 2018, we developed both ARDL and NARDL models and subsequently made predictions for January to December 2019. Figure 4 showcases the simulation and forecasting outcomes, while Table 4 delves into the predictive accuracy of each model. It was evident that the NARDL’s error metrics were consistently lower than the ARDL’s in both the simulation and prediction stages. Moreover, when comparing the forecasting performance with the commonly used GAM and ARIMA models, the NARDL model demonstrated lower forecasting error rates than those of both models (Table S4). Importantly, the sensitivity analysis revealed that the NARDL model also exhibited lower forecasting error rates compared to other models (Table S4). These results emphasize the effectiveness and suitability of the NARDL model in capturing the intricate interrelationships present in HFRS incidence data.

**Table 4:** Comparison of the fitting and forecasted abilities between ARDL and NARDL.

Models	Fitting part				Forecasting part			
	MAD	MAPE	RMSE	MER	MAD	MAPE	RMSE	MER
ARDL	21.484	0.238	30.814	0.175	32.485	0.477	42.583	0.520
NARDL	20.564	0.237	28.453	0.167	16.754	0.263	21.984	0.268

## Discussion

Growing evidence associates climatic parameters with HFRS^11,15,16^. However, the asymmetric relationships in both long and short terms have been underexplored. This study, utilizing the NARDL, is pioneering in decomposing climatic parameters into positive and negative partial sums to assess these effects on HFRS in Shandong. Our results underscore meteorological factors' dual role in influencing HFRS transmission both in long- and short-term contexts. The NARDL, incorporating these factors, depicted a more accurate dependence structure in HFRS than the ARDL, supporting the early predictive utility of combining meteorological factors and NARDL for HFRS risk.

There has been a decline in HFRS cases in Shandong during 2004–2019 (AAPC = − 9.568%), aligning with the broader trend observed across China^4^. Nonetheless, a few cities, such as Daqing, Songyuan, and Tonghua, have seen a minor uptick in recent years^11^. Government initiatives like vaccination, rodent control, public awareness campaigns, and environmental management have been crucial in driving this decline^4^. The pattern of HFRS cases shows two prominent peaks, one in spring and another in autumn, which corresponds with the seasonal pattern seen in both China and Korea^4,30^. This seasonality might be intimately tied to rodent population dynamics, human behavior, and environmental conditions^31,32^. One potential impact of seasonality on HFRS cases is the fluctuation in rodent populations throughout the year. Rodents are the primary reservoirs of hantaviruses, and their populations tend to increase during the warmer months when food and shelter are more abundant^31^. This increase in rodent populations can lead to a higher risk of human exposure to the virus, resulting in an uptick in HFRS cases during the spring and autumn^32^. Human behavior may also play a role in the seasonality of HFRS cases^31^. People tend to spend more time outdoors during the warmer months, increasing their chances of coming into contact with infected rodents or their droppings. Furthermore, agricultural activities such as farming and harvesting also peak during the spring and autumn months in Shandong, potentially increasing the risk of exposure to hantaviruses^32^.

Predicting HFRS epidemic based on meteorological factors is instrumental in shaping proactive responses, ensuring public safety, and optimizing resource utilization. This study found that the integration of meteorological factors into the NARDL model represents a significant advancement in the prediction of HFRS incidence. Compared to the commonly used ARDL model, the “autoregressive” term of NARDL is able to include delayed values of HFRS morbidity itself, the “nonlinear” aspect shows that the association of HFRS with meteorological parameters can be nonlinear, and the “distributed lag” implies that current observations of HFRS incidence are influenced by its past observations and past observations of meteorological parameters. These enable offering several advantages in modeling HFRS incidence series^20–23^: (1) asymmetry. This refers to cases where the impact of positive changes in weather factors might be different from the impact of negative changes; (2) short- and long-term dynamics. Including lagged values of variables in the model allows for the examination of both immediate and persistent impacts of weather factors, contributing to a more comprehensive analysis; (3) easy interpretation. NARDL allows for straightforward interpretation of coefficients, as it can directly capture the direction and magnitude of the effects of weather factors. This enhances the understanding of the relationship between variables and facilitates policy or decision-making; (4) enhanced model fit: the inclusion of nonlinear and asymmetric terms in NARDL improves the model fit by better capturing the underlying dynamics of the data, leading to more accurate and reliable predictions. These qualities equip the NARDL model to better consider the complex interactions between meteorological conditions and HFRS transmission dynamics, resulting in a more accurate and nuanced prediction of HFRS epidemic by use of the NARDL model. Therefore, it seems that the weather-integrated NARDL can be transferable to analyze and forecast HFRS epidemic in other regions and even for all similar phenomena, but it entails further validation.

Our findings revealed a significant and asymmetric impact of rainfall on HFRS, both in the short (WSR = − 2.419, *P* = 0.017) and long (WLR = − 2.697, *P* = 0.008) terms. From a long-term perspective, we observed a positive relationship despite no significance in the long-term coefficient (AP(+) = 0.664, *P* = 0.115; AP(−) = 0.251, *P* = 0.545). This finding concurs with a previous study^33^, which suggested a causal link between autumn crop production and HFRS. Precipitation affects vegetation growth and rodent food availability. Increased rainfall can lead to abundant food resources for rodents, causing an upsurge in their population, thus higher transmission rates of hantaviruses^18^. But, adopting a short-term view, we found a reverse association between AP(+) at 1–2 month delays (with coefficients of − 0.235, *P* = 0.018; − 0.198, *P* = 0.026, respectively) and HFRS, aligning with prior studies conducted in Shandong^34^, Anqiu^35^, Jiaonan^36^, together with Jiamusi and Qiqihar^37^. Notably, the majority of HFRS cases in China have occurred in low-lying regions and wetlands^37^. In such environments, heavy short-term rainfall serves to disrupt rodent nests and diminish rodent-human interactions due to reduced rodent activity and decreased human exposure over the long term^37^. However, our results diverge from a study in Heilongjiang that indicated no connection between rainfall and HFRS^38^. This discrepancy may be due to three potential reasons: (1) the earlier study employed a linear ARIMA model that failed to capture the complex relationship between these variables^38^; (2) the previous findings were derived from data spanning January 2001 to December 2009^38^, whereas our findings were based on data from January 2004 to December 2019, potentially resulting in divergent outcomes due to the different time periods analyzed; (3) the prior study solely gathered MT, MRH, and AP data^38^, without accounting for other meteorological confounding variables.

This study highlights a short-term asymmetric correlation between humidity and HFRS (WSR = − 2.515, *P* = 0.013), rather than a long-term asymmetry (WLR = − 1.81, *P* = 0.072). In the long term, we observed a positive relationship (MRH(+) = 9.045, *P* = 0.004; MRH(−) = 5.758, *P* = 0.066), matching well with the findings from other investigations^10,35^. Humidity can impact rodent behavior, such as their movement and nesting habits^11^. Specific humidity conditions may favor virus survival outside the host, increasing the risk of transmission^11^. Also, a prior study identified a temporal correlation between host densities in the third quarter and HFRS in the fourth quarter and revealed a positive link between MRH and host densities in the third quarter^39^. This consistency with our findings suggests that MRH could influence the densities of mites on hosts. In the short term, we observed a reverse relationship between HFRS and MRH(−) with a l-month lag (coefficient = − 1.552, *P* = 0.047), in line with earlier investigations^15,18^. As MRH is closely linked with AP, our study’s findings also align well with the outcomes seen in AP-related research^19^. This congruence could be explained by reduced rodent-human interaction, decreased rodent population density, and a more pronounced adverse effect of elevated MRH on hantavirus infectivity and stability^11,18^.

This study indicated long- (WLR = 2.561, *P* = 0.011) and short-run (WSR = 2.075, *P* = 0.04) asymmetries between ASH and HFRS (with coefficients of ASH(+) = 1.649, *P* = 0.018, ASH(−) = 2.107, *P* = 0.001, and ∆ASH(−) = 0.357, *P* = 0.04), consistent with earlier observational studies^17,35^. Sunlight may not directly affect rodent populations, but it has an indirect effect. Sunlight exposure is closely related to the ecological environment, particularly in humid areas, which are conducive to the survival and reproduction of rodents, leading to an increase in the rodent population, elevating the potential reservoirs of hantaviruses and thus raising the risk of HFRS transmission^11^. Besides, Shandong is the largest agricultural province in China, where suitable sunlight exposure often plays a significant role in agricultural production. Farmlands provide abundant food resources, attracting the aggregation of rodents. Crop cultivation and agricultural activities might disturb soil and vegetation, causing changes in rodent habitats, making them more likely to come into contact with humans and increasing the opportunities for HFRS transmission^11^.

A long-term symmetric negative correlation between MAP and HFRS was observed (MAP = − 1.727, *P* = 0.006), inconsistent with recent studies^16,40^. Plausible explanations are that^16,28,40,41^: (1) changes in MAP might influence rodent behavior, potentially impacting their interaction with humans or with each other. This could affect the transmission dynamics of the hantaviruses responsible for HFRS. (2) MAP can affect weather patterns and conditions, potentially impacting the habitats or breeding conditions of the rodents that are vectors for HFRS. A study indicated that elevated MAP levels are often linked to lower humidity^28,41^, these conditions are unfavorable for the survival and transmission of hantaviruses. (3) Changes in MAP often come with weather changes, which might alter human outdoor activities, influencing the likelihood of contact with infected rodents.

A negative correlation between increased MWV and HFRS (with coefficients of ∆MWV(+) = − 77.158, *P* < 0.001, ∆MWV(+) at a l-month lag = − 44.755, *P* = 0.007) was observed in the short term (WSR = − 4.594, *P* < 0.001), fitting well with a recent study in Shenyang^15^. Such an association can be attributed to several factors^17,28,41^. First, higher wind speeds can lead to better dispersion of aerosols and particles in the air, potentially reducing the concentration and longevity of infectious agents responsible for HFRS transmission. Second, increased wind can inhibit the survival and stability of viruses in the environment, making it more challenging for the causative agents of HFRS to persist. Lastly, higher wind speeds are often associated with improved ventilation and air circulation, which can dilute the concentration of pathogens and prevent their accumulation in specific areas. This, in turn, reduces the likelihood of contact between humans and the sources of infection. Besides, studies indicated that temperature can influence the breeding and viability of rodents, as well as the infectivity of hantaviruses^17,18,42,43^. Also, it can impact the behaviors of both rodent populations and human communities^11,18,43^. However, our study suggested an uncorrelation between them. This discrepancy may be because^40,42,44^: (1) prior studies used linear models such as generalized linear model and SARIMA, which cannot adequately capture the dynamic relationship between them; (2) meteorological factors exhibited great variation in different regions, thus further investigation was warranted locally, favoring the development of region-specific climate-based forecasting models.

The practical implications of our findings: by analyzing the long- and short-run asymmetric relationships between meteorological factors and HFRS, public health authorities in Shandong can better understand the intricate environmental conditions that are conducive to the spread of the disease. This information can then be used to develop early warning systems that alert communities to the potential for an outbreak of HFRS, allowing for timely intervention and prevention measures^38,45^. In addition, by understanding how changes in meteorological conditions affect the incidence of HFRS, authorities can implement targeted interventions such as rodent control measures, public education campaigns, and vaccination programs to reduce the risk of transmission. This proactive approach can help to prevent outbreaks of HFRS and minimize the burden of the disease on affected communities^46^. Also, the findings can prioritize surveillance and control efforts in high-risk HFRS areas, ensuring that resources are directed where they are most needed. This targeted approach can help to maximize the impact of prevention strategies and improve the overall effectiveness of public health interventions.

Our study also has some limitations. First, it is inevitable to encounter under-reporting or under-diagnosis issues in a passive monitoring system. Second, being an ecological trend study, it does not allow for the exploration of individual-level relationships or the inference of causal effects. Third, a more detailed temporal analysis could have been achieved with daily or weekly data, but their unavailability hinders further investigation. Fourth, this study only considers the long and short-term asymmetric independent effects of meteorological factors on HFRS. Further research is needed to explore the complex the long and short-term asymmetric interactions of pollution and climatic factors on HFRS and to develop comprehensive strategies to protect the ecological barrier of HFRS for mitigating the impact of HFRS on public health^12,15^. Fifth, the current findings are based on data from Shandong, it is worth further exploring whether these discoveries can be generalized to other regions with different climate conditions. Lastly, we did not account for the impact of unmeasured confounders such as geographic and socioeconomic factors, population density, and host susceptibility.

## Conclusions

Taken together, our findings highlight the significant and potentially asymmetric and/or symmetric roles of weather factors in the long- and short-term HFRS incidence. Integrating meteorological variables into public health intervention plans appears crucial, especially in the context of global climate change. The NARDL proves more suitable for capturing the dynamic epidemic structure of HFRS incidence compared to the ARDL. Weather-integrated NARDL prediction model for HFRS is a promising approach in the realm of public health, offering a proactive means to anticipate, prepare for, and combat disease epidemic. By understanding and harnessing the relationships between weather patterns and HFRS dynamics, we can better protect communities and reduce this disease burden.

### Supplementary Information

Supplementary Information.

## Data Availability

All data for this work are presented in the results and conclusions or please contact the corresponding author on the reproducibility of this work.
