# Supplementary material for: Asymmetric impact of climatic parameters on hemorrhagic fever with renal syndrome in Shandong using a nonlinear autoregressive distributed lag model
Source: Sci Rep. 2024 Apr 28;14:9739. doi: 10.1038/s41598-024-58023-9 (PMC11056385; doi:10.1038/s41598-024-58023-9)
Supplement: Supplementary file 1 — Supplementary Information. [file 41598_2024_58023_MOESM1_ESM.docx]

**Supplementary material：****Asymmetric impact of climatic parameters on** **hemorrhagic fever with renal syndrome in Shandong using a nonlinear autoregressive distributed lag model**

Yongbin Wang^1^, Ziyue Liang^1^, Siyu Qing^1^, Yue Xi^1^, Chunjie Xu^2^, Fei Lin^1^

^1^ Department of Epidemiology and Health Statistics, School of Public Health, The First Affiliated Hospital of Xinxiang Medical University, No. 601 Jinsui Road, Hongqi District, Xinxiang, Henan Province 453003, People’s Republic of China

^2^ Beijing Key Laboratory of Antimicrobial Agents/Laboratory of Pharmacology, Institute of Medicinal Biotechnology, Chinese Academy of Medical Sciences & Peking Union Medical College, Beijing, 100050, China

Corresponding author: Yongbin Wang ([wybwho@163.com](mailto:wybwho@163.com)) and Fei Lin (linfeixixi@aliyun.com)

**Criteria used for selecting the ARDL and NARDL models**

In the field of time series analysis, selecting the appropriate model for analysis is crucial in order to ensure accurate and reliable results. When it comes to time series data, the ARDL and NARDL models are commonly used for estimating both long-run and short-run relationships among variables[^1-4^](#_ENREF_1).

The ARDL model is able to be a popular choice for estimating long-run and short-run relationships in time series data simultaneously because it allows for the inclusion of both stationary and non-stationary variables in the same model. This is important when dealing with time series data, as many variables are non-stationary and exhibit long-term trends in time series analysis. The ARDL model is also flexible in terms of the lag structure, allowing to specify the appropriate number of lags based on the data and the underlying the nature of data (e.g., the incubation period of infectious diseases). One of the key criteria for selecting the ARDL model is the presence of cointegration among the variables of interest[^3^](#_ENREF_3)^,^[^5^](#_ENREF_5). Cointegration implies a long-run relationship between variables, meaning that they move together over time despite short-term fluctuations. The ARDL model is well-suited for estimating such relationships and can provide insights into the dynamics of the variables both in the long and term run.

In contrast, the NARDL model is specifically designed to capture nonlinear relationships among variables in time series data. Nonlinear relationships are common in time series data, as variables may not move in a linear fashion but rather exhibit complex patterns and interactions. The NARDL model allows to capture these nonlinearities and estimate the long-run and short-run relationships simultaneously. When selecting the NARDL model, researchers should consider the nature of the relationships among variables and whether linear models are sufficient to capture the dynamics of the data. If there is evidence of nonlinearities or threshold effects in the data, the NARDL model may be more appropriate for the analysis[^1^](#_ENREF_1). Additionally, the NARDL model can provide insights into the asymmetries in the relationships among variables, which may not be captured by linear models.

In terms of applicability to the study's specific objectives, researchers should carefully consider the research questions and hypotheses they are trying to test. If the objective is to estimate both long-run and term-run relationships among variables and investigate the dynamics of the data over time, the ARDL model may be more suitable[^3^](#_ENREF_3). On the other hand, if the objective is to explore nonlinear relationships and capture complex patterns in the data, the NARDL model may be a better choice[^1^](#_ENREF_1). Furthermore, researchers should also consider the data requirements and assumptions of each model when selecting the appropriate model for analysis. The ARDL model requires the variables to be integrated of the same order[^3^](#_ENREF_3), while the NARDL model does not have this restriction. Researchers should assess whether the data meet the assumptions of the models and choose the one that best fits the data and the research objectives.


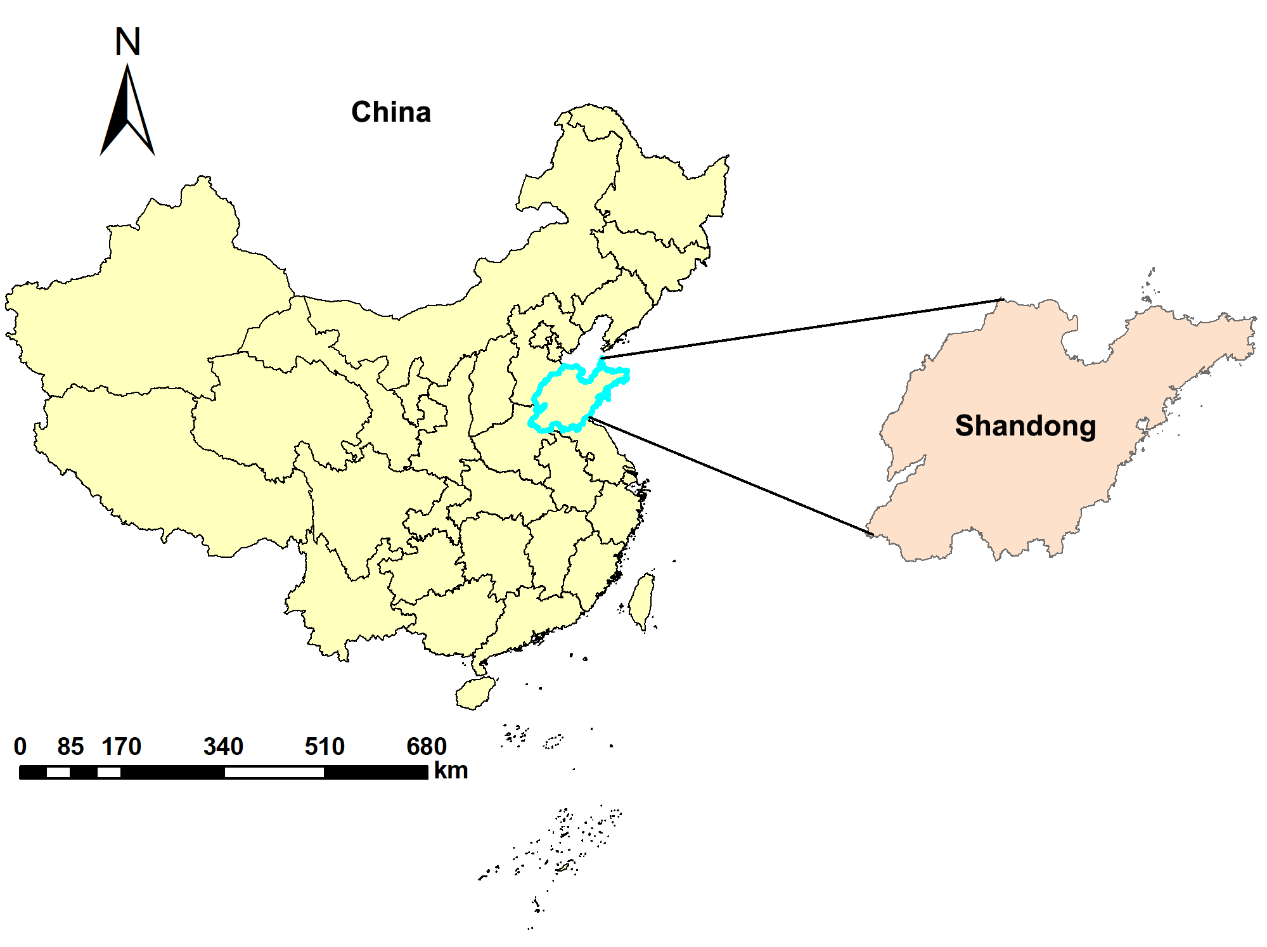


Figure S1. Geographical distribution of Shandong (Created by ArcGIS 10.8). Note: The basic geographic information data of China were downloaded from the Resource and Environmental Science and Data Center (Available at: https://www.resdc.cn/Default.aspx. Accessed on 23 February, 2024).

Data Collection and Preprocessing

transform the data into a suitable format for analysis, ensuring compatibility with the model requirements

check for missing values, outliers, and inconsistencies in the data

gather HFRS cases in Shandong Province and relevant meteorological variables

Define the NARDL model structure and select the appropriate functional form for the nonlinear relationship between meteorological factors and HFRS incidence

Model estimation and parameter test are used to determine the parameters, reliability and validity of the model

Use the NARDL model to predict the future incidence of HFRS and explain the results of the model

Provide a scientific basis for the prevention and control of HFRS

Figure S2. Overall methodological flow chart.


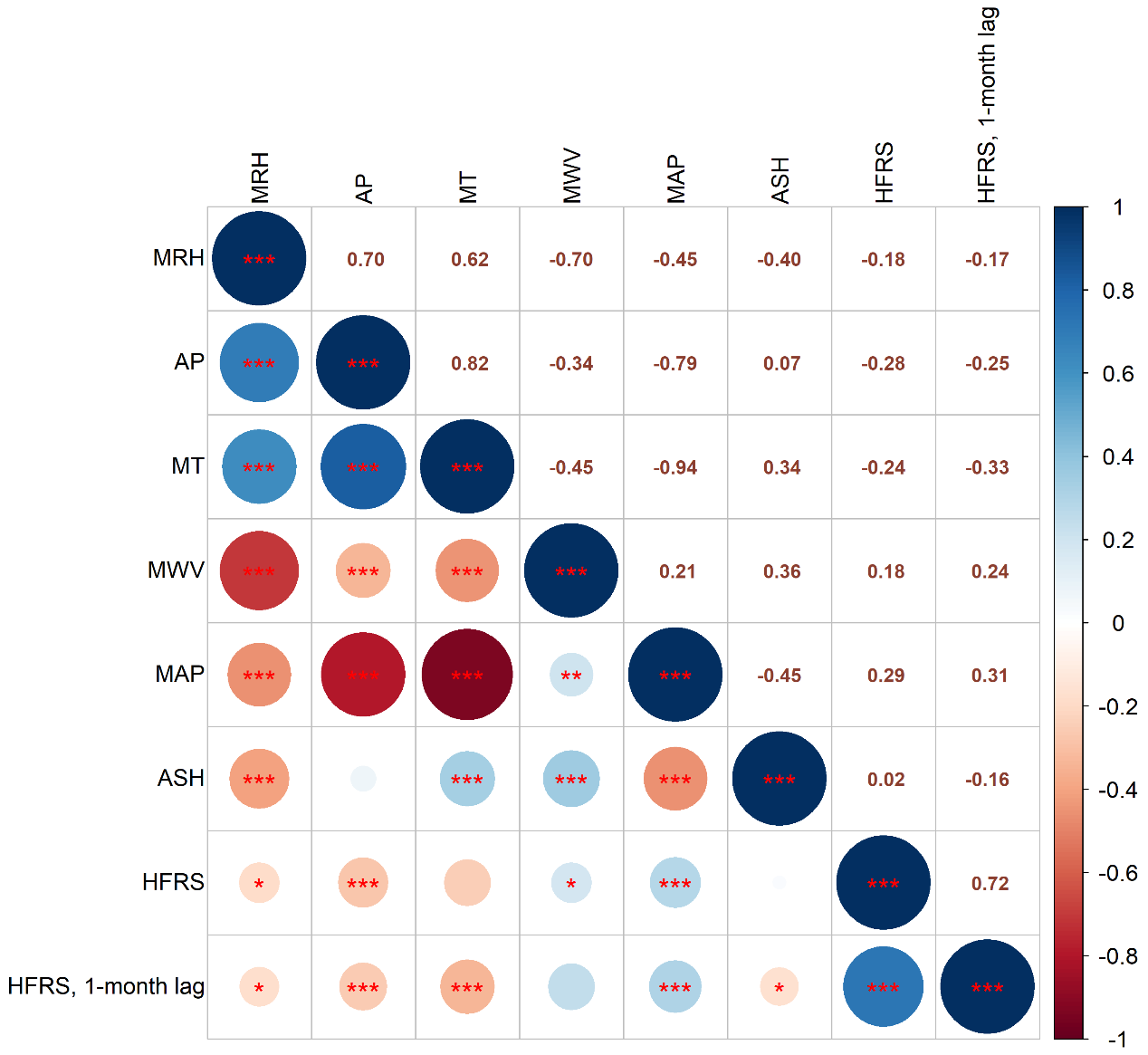


**Figure S3.** Spearman’s correlation between variables. As shown, there was a correlation greater than 0.9 between MT and MAP, showing a presence of strong collinearity between them.


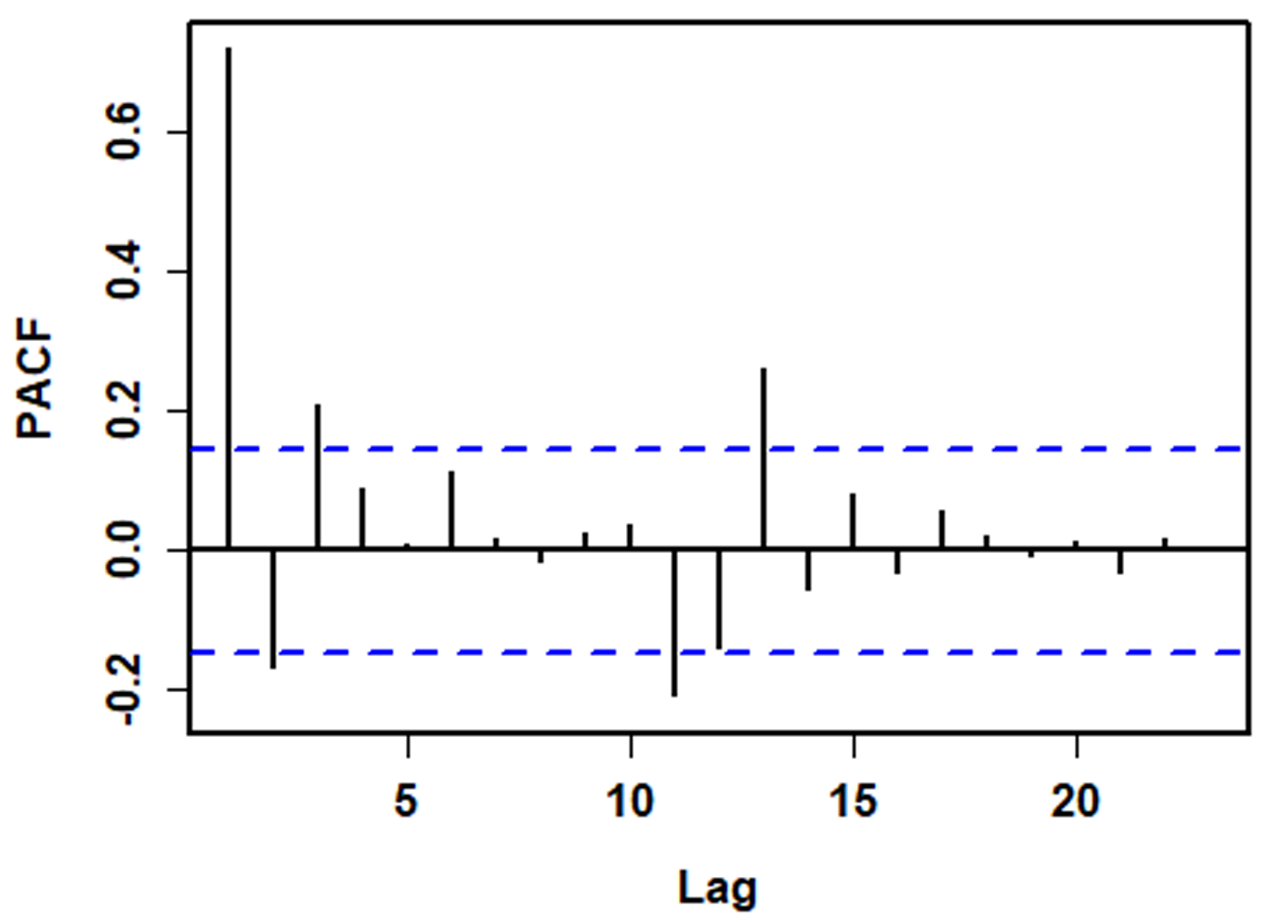


**Figure S4.** Partial autocorrelation function (PACF) plot for the HFRS incidence series. A local maximum value at 1-month lag was found, indicating the first-order autocorrelation between HFRS incidence series that may be included in the model.

**Figure S5.** Akaike information criterion (AIC) of top 20 NARDL models.

**Figure S6.** Akaike information criterion (AIC) of top 20 ARDL models.

**Table S1** The annualized statistical description for the monthly HFRS and climatic data in Shandong between 2004 and 2019

| Variable | Mean | S.D. | Min | P_25_ | P_50_ | P_75_ | Max | *P^*^* | VIF | VIF^a^ | VIF^b^ |
| --- | --- | --- | --- | --- | --- | --- | --- | --- | --- | --- | --- |
| HFRS cases | 119.15 | 94.45 | 23.00 | 53.75 | 82.00 | 144.50 | 465.00 | <0.001 | — | — | — |
| MRH | 65.26 | 9.42 | 44.83 | 57.99 | 64.66 | 72.55 | 85.38 | 0.003 | 8.29 | 8.04 | 5.83 |
| AP | 57.28 | 67.10 | 0.39 | 10.42 | 29.12 | 76.16 | 345.62 | <0.001 | 3.88 | 3.25 | 3.84 |
| MT | 13.47 | 9.73 | -4.37 | 4.49 | 14.65 | 22.55 | 27.52 | <0.001 | 20.97 | 8.51 | — |
| MWV | 2.68 | 0.43 | 1.88 | 2.37 | 2.63 | 2.93 | 3.72 | <0.001 | 2.61 | 2.13 | 2.03 |
| MAP | 1003.76 | 8.072 | 989.55 | 995.97 | 1004.35 | 1010.95 | 1018.00 | <0.001 | 13.77 | — | 5.59 |
| ASH | 191.61 | 40.34 | 71.17 | 162.81 | 193.08 | 219.30 | 299.18 | 0.694 | 5.59 | 5.58 | 3.71 |
| HFRS cases,  1-month lag | — | — | — | — | — | — | — | — | 1.20 | 1.20 | 1.18 |

*^*^*Shapiro-Wilk normality test, a Removal of the MAP variable, b Removal of the MT variable.

**Table S2.** Estimated parameters for the selected best possible NARDL (1, 0, 2, 3, 0, 2, 0, 0, 1, 1, 0)

| Variables | Coefficient | Std. Error | t-Statistic | *P* value |
| --- | --- | --- | --- | --- |
| HFRS, 1-month lag | 0.6808 | 0.0315 | 21.6392 | <0.001 |
| MRH (+) | 2.8870 | 0.9728 | 2.9676 | 0.0035 |
| MRH (-) | -1.7322 | 1.0114 | -1.7127 | 0.0887 |
| MRH (-), 1-month lag | 2.0181 | 0.9111 | 2.2149 | 0.0282 |
| MRH (-), 2-month lag | 1.5519 | 0.7733 | 2.0069 | 0.0465 |
| AP (+) | -0.0423 | 0.0912 | -0.4633 | 0.6438 |
| AP (+), 1-month lag | 0.0190 | 0.1478 | 0.1283 | 0.8981 |
| AP (+), 2-month lag | 0.0373 | 0.1021 | 0.3648 | 0.7157 |
| AP (+), 3-month lag | 0.1978 | 0.0883 | 2.2414 | 0.0264 |
| AP (-) | 0.0801 | 0.1322 | 0.6056 | 0.5457 |
| MWV (+) | -77.1582 | 16.2367 | -4.7521 | 0.0000 |
| MWV (+), 1-month lag | 56.8620 | 19.7783 | 2.8750 | 0.0046 |
| MWV (+), 2-month lag | 44.7554 | 16.4791 | 2.7159 | 0.0074 |
| MWV (-) | 21.3737 | 14.8689 | 1.4375 | 0.1526 |
| ASH (+) | 0.4688 | 0.1860 | 2.5201 | 0.0127 |
| ASH (-) | 0.3570 | 0.1720 | 2.0751 | 0.0396 |
| ASH (-), 1-month lag | 0.3156 | 0.1498 | 2.1061 | 0.0368 |
| MAP (+) | -2.1649 | 1.8195 | -1.1898 | 0.2359 |
| MAP (+), 1-month lag | 3.0191 | 2.0140 | 1.4991 | 0.1359 |
| MAP (-) | 2.5567 | 1.8150 | 1.4087 | 0.1609 |
| @MONTH=1 | 18.0889 | 10.3493 | 1.7478 | 0.0824 |
| @MONTH=2 | 43.1967 | 10.7669 | 4.0120 | 0.0001 |
| @MONTH=3 | 65.2539 | 15.1054 | 4.3199 | 0.0000 |
| @MONTH=4 | 57.6051 | 23.4899 | 2.4523 | 0.0153 |
| @MONTH=5 | 59.5924 | 33.7281 | 1.7668 | 0.0792 |
| @MONTH=6 | 38.2445 | 41.7524 | 0.9160 | 0.3611 |
| @MONTH=7 | 21.4783 | 49.0111 | 0.4382 | 0.6618 |
| @MONTH=8 | 31.5536 | 50.7503 | 0.6217 | 0.5350 |
| @MONTH=9 | 56.2831 | 40.6779 | 1.3836 | 0.1684 |
| @MONTH=10 | 189.6971 | 27.1040 | 6.9988 | <0.001 |
| @MONTH=11 | 155.5603 | 14.2367 | 10.9267 | <0.001 |
| R-squared | 0.9005 | Mean dependent variable | | 114.4415 |
| Adjusted R-squared | 0.8815 | S.D. dependent variable | | 89.2795 |
| S.E. of regression | 30.7322 | Akaike info criterion | | 9.8381 |
| Sum squared residual | 148281.7000 | Schwarz criterion | | 10.3718 |
| Log likelihood | -893.7806 | Hannan-Quinn criterion | | 10.0543 |
| Durbin-Watson statistic | 1.7515 |  |  |  |

**Table S3.** Estimated parameters for the selected best possible ARDL(1, 0, 0, 1, 0, 0)

| Variables | Coefficient | Std. Error | t-Statistic | *P* value |
| --- | --- | --- | --- | --- |
| HFRS, 1-month lag | 0.8410 | 0.0381 | 22.0968 | <0.001 |
| MRH | 1.4787 | 0.8495 | 1.7407 | 0.0835 |
| AP | -0.0226 | 0.0908 | -0.2487 | 0.8039 |
| MWV | -16.0670 | 11.8461 | -1.3563 | 0.1768 |
| MWV, 1-month lag | 22.3072 | 11.2882 | 1.9762 | 0.0497 |
| ASH | 0.5131 | 0.1663 | 3.0849 | 0.0024 |
| MAP | -0.2747 | 0.0779 | -3.5281 | 0.0005 |
| @MONTH=1 | 58.9660 | 13.7382 | 4.2921 | <0.001 |
| @MONTH=2 | 101.5681 | 14.3085 | 7.0984 | <0.001 |
| @MONTH=3 | 103.0874 | 17.1715 | 6.0034 | <0.001 |
| @MONTH=4 | 74.7608 | 19.7369 | 3.7879 | 0.0002 |
| @MONTH=5 | 59.7171 | 23.9986 | 2.4884 | 0.0138 |
| @MONTH=6 | 39.3701 | 22.7550 | 1.7302 | 0.0854 |
| @MONTH=7 | 34.3471 | 29.2563 | 1.1740 | 0.2420 |
| @MONTH=8 | 39.5301 | 31.3207 | 1.2621 | 0.2086 |
| @MONTH=9 | 78.7949 | 22.7828 | 3.4585 | 0.0007 |
| @MONTH=10 | 231.5579 | 18.5211 | 12.5024 | <0.001 |
| @MONTH=11 | 177.0035 | 13.4250 | 13.1846 | <0.001 |
| R-squared | 0.8729 | Mean dependent variable | | 118.4241 |
| Adjusted R-squared | 0.8604 | S.D. dependent variable | | 94.1625 |
| S.E. of regression | 35.1761 | Akaike info criterion | | 10.0481 |
| Sum squared residual | 214063.3000 | Schwarz criterion | | 10.3546 |
| Log likelihood | -941.5947 | Hannan-Quinn criterion | | 10.1723 |
| Durbin-Watson statistic | 1.8365 |  |  |  |

**Table S4** Comparison of the fitting and forecasted abilities of NARDL with ARDL, GAM, and ARIMA

| Models | Fitting part | | | |  | Forecasting part | | | |  | |
| --- | --- | --- | --- | --- | --- | --- | --- | --- | --- | --- | --- |
|  | MAD | MAPE | RMSE | MER |  | MAD | MAPE | RMSE | | MER | |
| **12 holdout data forecasting** | | | | | | | | | | | |
| ARDL | 21.484 | 0.238 | 30.814 | 0.175 |  | 32.485 | 0.477 | 42.583 | | 0.520 | |
| NARDL | 20.564 | 0.237 | 28.453 | 0.167 |  | 16.754 | 0.263 | 21.984 | | 0.268 | |
| GAM | 31.409 | 0.356 | 42.407 | 0.256 |  | 20.927 | 0.326 | 26.672 | | 0.335 | |
| ARIMA(0,1,2)(1,1,0)_12_ | 23.387 | 0.215 | 40.522 | 0.190 |  | 66.549 | 0.875 | 110.202 | | 1.066 | |
| **24 holdout data forecasting** | | | | | | | | | | |  |
| ARDL | 20.533 | 0.231 | 29.751 | 0.165 |  | 31.494 | 0.409 | 42.561 | 0.384 | |  |
| NARDL | 19.297 | 0.209 | 27.447 | 0.155 |  | 23.128 | 0.341 | 29.563 | 0.282 | |  |
| GAM | 35.952 | 0.412 | 48.453 | 0.289 |  | 28.078 | 0.399 | 47.202 | 0.343 | |  |
| ARIMA(1,1,1)(0,1,1)_12_ | 23.529 | 0.206 | 38.855 | 0.189 |  | 39.414 | 0.554 | 57.584 | 0.481 | |  |

## References

1 Shin, Y., Yu, B. & Greenwoodnimmo, M. Modelling Asymmetric Cointegration and Dynamic Multipliers in a Nonlinear ARDL Framework. *Social Science Electronic Publishing*, 281-314 (2014).

2 Bakry, W., Nghiem, X.-H., Farouk, S. & Vo, X. V. Does it hurt or help? Revisiting the effects of ICT on economic growth and energy consumption: A nonlinear panel ARDL approach. *Economic Analysis and Policy* **78**, 597-617 (2023).

3 Li, J. F. & Lin, Z. X. The impact of sales tax on economic growth in the United States: an ARDL bounds testing approach. *Applied Economics Letters* **22**, 1262-1266, doi:10.1080/13504851.2015.1023933 (2015).

4 Samantaraya, A. & Patra, S. K. Determinants of Household Savings in India: An Empirical Analysis Using ARDL Approach. *Economics Research International* **2014**, 1-8.

5 Srinivasan, P., Kumar, P. K. S. & Ganesh, L. Tourism and Economic Growth in Sri Lanka: An ARDL Bounds Testing Approach. *Romanian Economic Journal* **3**, 397-405 (2012).
